# Ideal P2Y12 Inhibitor in Acute Coronary Syndrome: A Review and Current Status

**DOI:** 10.3390/ijerph19158977

**Published:** 2022-07-23

**Authors:** Akshyaya Pradhan, Aashish Tiwari, Giuseppe Caminiti, Chiara Salimei, Saverio Muscoli, Rishi Sethi, Marco Alfonso Perrone

**Affiliations:** 1 Department of Cardiology, King George’s Medical University, Lucknow 226003, Uttar Pradesh, India; akshyaya33@gmail.com (A.P.); aashishtiwari@live.com (A.T.); drrishisethi1@gmail.com (R.S.); 2 Cardiology Rehabilitation Unit, S. Raffaele IRCCS, 00163 Rome, Italy; giuseppe.caminiti@sanraffaele.it; 3 Department of Cardiology and CardioLab, University of Rome Tor Vergata, 00133 Rome, Italy; chiara.salimei@gmail.com (C.S.); saveriomuscoli@gmail.com (S.M.)

**Keywords:** acute coronary syndrome, dual antiplatelet therapy, prasugrel, ticagrelor, cangrelor, P2Y12 inhibitors

## Abstract

Dual antiplatelet therapy (DAPT) has remained the cornerstone for management of acute coronary syndrome (ACS) over the years. Clopidogrel has been the quintessential P2Y12 receptor (platelet receptor for Adenosine 5′ diphosphate) inhibitor for the past two decades. With the demonstration of unequivocal superior efficacy of prasugrel/ticagrelor over clopidogrel, guidelines now recommend these agents in priority over clopidogrel in current management of ACS. Cangrelor has revived the interest in injectable antiplatelet therapy too. Albeit the increased efficacy of these newer agents comes at the cost of increased bleeding and this becomes more of a concern when combined with aspirin. Which P2Y12i is superior over another has been intensely debated over last few years after the ISAR-REACT 5 study with inconclusive data. Three novel antiplatelet agents are already in the pipeline for ACS with all of them succeeding in phase II studies. The search for an ideal antiplatelet remains a need of the hour for optimal reduction of ischemic events in ACS.

## 1. Introduction

The term dual antiplatelet therapy (DAPT) has been used to specifically refer to combination therapy of aspirin and a P2Y12 receptor inhibitor (clopidogrel, prasugrel, ticagrelor, or cangrelor). In acute coronary syndromes, a 12-month DAPT is recommended according to current American Heart Association (AHA) guidelines except in special scenarios [1,2,3]. These guidelines also recommended preferred use of prasugrel/ticagrelor in acute coronary syndrome (ACS) patients treated by percutaneous coronary intervention (PCI). In medically managed patients also ticagrelor has found a place over clopidogrel because of its superior platelet inhibition over clopidogrel [3,4]. However which P2Y12 inhibitor is superior among prasugrel, ticagrelor, and cangrelor has been debated over the years with an inconclusive mandate. In the landmark ISAR REACT-5 trial, it was revealed that in ACS patients with or without ST-segment elevation, treatment with prasugrel as compared to ticagrelor significantly reduced major adverse cardiac events (MACEs) without an increase in major bleeding [5]. However, after critical analysis of trial data this benefit of prasugrel over ticagrelor appears inconclusive [6,7].

The platelet P2Y12 receptor has a key role in thrombus formation during ACS. It is a G-protein coupled receptor on platelet for which adenosine diphosphate is the ligand. The ADP binding leads to shape change, platelet aggregation, secretion of dense granules, stabilization of platelet aggregates, and last but not the least amplification of platelet responses all culminating in enhancement of the prothrombotic effect [8]. Hence, underscoring the central role of P2Y12 inhibitors for reduction of ischemic events during ACS.

In this review, we discuss the rise of clopidogrel in clinical practice, its pitfalls, and the need for more potent P2Y12 inhibitors. We then describe the clinical evidence supporting the use of newer agents and critically analyze data to support preferential use of one agent over other. We try to rationalize the use of different P2Y12 inhibitors in various clinical scenarios based on contemporary literature. Finally, we touch upon the recommendations from guidelines across the globe and discuss few upcoming novel anti platelet agents poised to enter the clinical scenario.

## 2. Evolution of Guidelines over the Years and Journey of Clopidogrel

**Figure 1:**
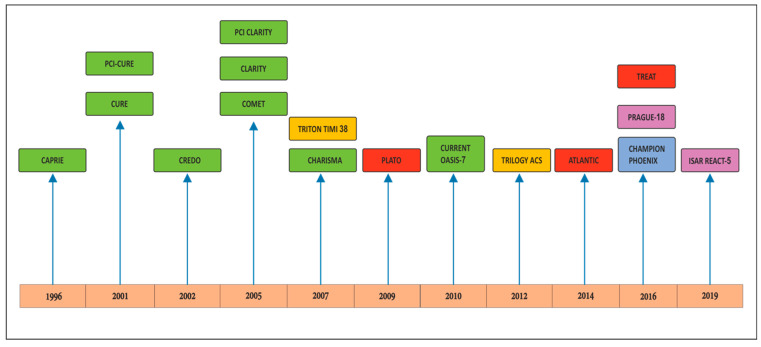
Timeline of P2Y12 inhibitor trials over the past 25 years. The green boxes represent clopidogrel trials, the yellow boxes prasugrel trials, and the red boxes contain ticagrelor trials. The blue box represents the cangrelor trial while the purple ones represent studies comparing ticagrelor versus prasugrel.

Clopidogrel is the seminal P2Y12 inhibitor available worldwide. The drug has had the maximum patient data and safety records over the years. There is vast experience of over two decades with the drug. The clopidogrel journey started with CAPRIE, CURE, CREDO, and COMMIT trials which established its use in NSTEMI ACS and post PCI (percutaneous coronary intervention) patients [9,10,11,12,13]. CLARITY AND PCI-CLARITY established the role of clopidogrel in STEMI patients [14,15] (Figure 1, Table 1). However, there are several limitations of clopidogrel use in ACS. First of all, it has slow onset of action (3–8 h), moderate overall levels of platelet inhibition with average inhibition of platelet aggregation (IPA) ~50%, and finally even this moderate inhibition has high variability response within a population (4–34% with very low levels of platelet inhibitions). In fact with a standard loading dose of 300 mg, max platelet inhibition occurs only after 6 h. Apart from slow onset of action, high inter-patient variability in platelet inhibition with clopidogrel makes it a poor choice as an effective antiplatelet drug in the current intervention era [16]. The current OASIS 7 study suggested that even doubling the loading and maintenance dose did not make any difference [17]. Even the platelet function test (PFT) guided tailoring and had no significant effect (Table 2) [18,19,20]. In post thrombolysis patients large-scale randomized controlled trials (RCTs) have shown that dual antiplatelet therapy with aspirin and clopidogrel reduces major cardiovascular events in fibrinolytic-treated STEMI patients. Data from the CLARITY (TIMI) 28 trial suggested that adding clopidogrel to fibrinolysis is safe and effective in patients <75 years of age [14]. Current guidelines recommend clopidogrel in addition to aspirin for 1 year in patients treated with fibrinolytic agents [1,2,3,4]. Figure 1 depicts the timeline of major P2Y12 inhibitor trials over the past 25 years. 

## 3. Journey of Newer P2Y12i: Prasugrel, Ticagrelor, and Cangrelor

Prasugrel is a 3rd generation thienopyridine with an efficient generation of active metabolite. It provides rapid, potent, and consistent IPA (inhibition of platelet aggregation) [1,2,3]. Prasugrel as compared to clopidogrel is more potent, more rapid in onset, has more efficient generation of its active metabolite and more consistent inhibition of platelet aggregation (Table 3). Prasugrel is also effective for clopidogrel non-responders. The prasugrel journey started with the TRITON TIMI 38 trial which established its role in acute coronary syndrome patients undergoing PCI [21]. The primary endpoint of the study was a combination of CV death, MI, and stroke at the end of 2 years, where prasugrel showed a significant benefit (9.9% vs. 12.1%, *p* = 0.0004, number needed to treat = 46). Prasugrel also showed a major benefit in stent thrombosis (1.1% vs. 2.4%, *p* < 0.0001). All of the above benefits in the study came at a cost of increase in major bleeding (2.4% vs. 1.8%, *p* = 0.03) and no net clinical benefit in three subgroups of prior stroke/TIA, age > 75 years, and weight < 60 kg [21]. Prasugrel has also been studied in medically managed non-ST-segment elevation NSTE-ACS patients for primary end point of CV death, MI, or stroke in the TRILOGY ACS trial. The results of TRILOGY ACS demonstrated that outcomes (including major bleeding) were similar in the overall population, including the elderly. It concluded that among medically treated patients with NSTE-ACS, prasugrel did not reduce adverse outcomes compared with clopidogrel [22]. In the invasive approach group of NSTE-ACS, the ACCOAST trial concluded that pretreatment with prasugrel is not associated with superior ischemic outcomes in patients scheduled to undergo an invasive strategy with a significantly higher bleeding risk [23]. Therefore, prasugrel definitely reduced CV events including stent thrombosis in a selective population of STEMI and NSTEMI patients going for an invasive approach with a risk of increase bleeding. It is also established that prasugrel should not be given in medically managed acute coronary syndrome, a post thrombolysis setting, or in prior stroke/TIA. Its dose is supposed to be reduced in elderly and in low-body-weight patients. In a post thrombolysis setting, there is scarcity of published data regarding the use of prasugrel. 

Ticagrelor has a different chemical structure from clopidogrel and prasugrel. It is not a prodrug, so does not require metabolic activation [1,2]. More importantly, its binding to the target P2Y12 receptor is reversible so recovery of platelet function does not depend on generation of new platelets and it results in faster offset of platelet inhibition than clopidogrel. Data also suggest that clinical factors and the CYP2C19 variants do not significantly impact the outcomes in patients treated with ticagrelor. In brief, clopidogrel is a prodrug which is metabolized in the active form by a two-step enzymatic process in liver catalyzed by the cytochrome p450 enzyme which is encoded by the CYP2C19 gene [24]. Polymorphisms in the expression of this gene’s alleles leads to variations in metabolism of clopidogrel leading to inter-individual variation in therapeutic drug levels. Of note, the CYP2C19*2 and *3 alleles are categorized as poor metabolizers with low or absent enzymatic activity. Such variants have been associated with adverse cardiovascular outcomes including stent thrombosis.

Ticagrelor also achieved greater inhibition of platelet aggregation (IPA) in clopidogrel non-responders treated with ticagrelor compared with clopidogrel [25]. Ticagrelor has been established as a potent P2Y12 inhibitor over the years (Table 3). The PLATO trial, a landmark trial, studied prasugrel vs. clopidogrel in STEMI undergoing primary PCI and moderate to high risk NSTE-ACS undergoing PCI for primary end point of CV death, MI, and stroke. In the above study, the composite benefit of ticagrelor was seen as early as 30 days and this benefit continued to grow over 1 year [26]. 

Therefore, ticagrelor hits the sweet spot as it retains all advantages of prasugrel over clopidogrel and has the benefit of reversibility—can be stopped 3 days before surgery and no anatomical or clinical contraindications. On the downside, ticagrelor has twice daily dosing, higher cost, and dyspnea as significant side effects leading to discontinuation of the drug. Ticagrelor-induced dyspnea is reported in up to 20% of patients (range 5–20%); however, in many of them this dyspnea spontaneously improved within 3 days and some of them tolerate this dyspnea with appropriate counselling. In fact, dyspnea after ticagrelor has been linked to adenosine-dependent and -independent mechanism of ticagrelor. This effect is additional to P2Y12 receptor inhibition in platelets [27]. Interestingly, the dyspnea can be attenuated by lowering the long-term maintenance dose as seen with 60 mg BD dosing utilized in the PEGASUS-TIMI 54 study [28]. The PLATO trial included only patients undergoing primary PCI and patients who received fibrinolytic therapy in the preceding 24 h were excluded. Therefore, evidence on the longer term effects of ticagrelor in patients with STEMI treated with fibrinolytic therapy was lacking till the TREAT trial wherein patients who received fibrinolytic therapy for STEMI were randomized to delayed ticagrelor (*n* = 1913) versus clopidogrel (*n* = 1886). Patients were randomized a median of 11 h after fibrinolysis and 90% had been pretreated with clopidogrel. The TREAT trial studied combined CV death, MI, stroke, severe recurrent ischemia, TIA, or other arterial thrombotic events at 12 months. The trial concluded that among patients <75 years of age who were treated with fibrinolysis for STEMI, delayed administration of ticagrelor was noninferior to clopidogrel. There was no excess of major bleeding, fatal bleeding, or intracranial bleeding with ticagrelor vs. clopidogrel [29]. Pooled analysis of combined TREAT and PLATO suggests a reduction in MACE with no statistical heterogeneity evident between trials. Meta-analysis of 3 RCTs of 3999 patients comparing ticagrelor versus clopidogrel after fibrinolytic therapy demonstrated that STEMI patients treated with post-fibrinolysis ticagrelor experience similar short-term rates of bleeding and ischemic events as those who continued clopidogrel therapy [30]. There are ongoing studies of upfront therapy with ticagrelor in patients undergoing fibrinolytic therapy.

In both prasugrel and ticagrelor, ischemic benefits outweigh bleeding risk. The number needed to treat is 46 in prasugrel and 53 in ticagrelor while the number needed to harm is 167 in both prasugrel and ticagrelor. In a comparison of prehospital and in-hospital administration of ticagrelor in patients of STEMI, the ATLANTIC trial concluded that ticagrelor appeared to be safe but did not improve pre-PCI coronary reperfusion [31].

Cangrelor is an intravenous direct reversible, short-acting P2Y12 receptor blocker (non-thienopyridine adenosine triphosphate analogue) that has been evaluated in the setting of STEMI and also in elective PCI settings. This agent can be used in STEMI patients in cardiogenic shock and in patients where oral intake of the P2Y12 inhibitor is difficult. Though initial trials (CHAMPION PLATFORM, CHAMPION PCI) failed to show clinical superiority over clopidogrel in ACS/CCS patients undergoing PCI, a third trial, CHAMPION PHOENIX demonstrated benefit at end of 48 h in the composite primary efficacy endpoint (death, MI, ischemia-driven revascularization, or stent thrombosis) as compared to clopidogrel (4.7 versus 5.9 percent, OR 0.78, 95% CI 0.66–0.93). There was no significant difference in the severe or life-threatening bleeding at the end of 48 h [32,33]. A pooled analysis of these three trials showed that cangrelor reduced peri-procedural ischemic complications at the expense of increased bleeding [34]. Cangrelor was approved by the United States Food and Drug Administration (USFDA) in 2015 as an adjunct to PCI in patients who have not been treated with a P2Y12 platelet inhibitor and who are not being given a Gp IIb/IIIa inhibitor. Due to its proven efficacy in preventing intra-procedural and post-procedural stent thrombosis in P2Y12 receptor inhibitor-naïve patients, cangrelor may be considered on a case-by-case basis in P2Y12 receptor inhibitor-naïve NSTE-ACS patients undergoing PCI [35,36]. Table 3 enumerates the salient features of the major P2Y12 inhibitors in vogue. 

**Table 3:** Comparison of pharmacological characteristics of major P2Y12 receptor antagonists.

Characteristics	Clopidogrel	Prasugrel	Ticagrelor	Ellinogrel	Cangrelor	Selatogrel	References
Chemical class	Thienopyridine	Thienopyridine	Cyclopentyl-triazolo-pyrimidine	-	Nonthienopyridine adenosine triphosphate analogue	2-phenylpyrimidine-4-carboxamide analogue	[1,2,3,34,35]
Receptor blockage	Irreversible	Irreversible	Reversible	Reversible	Reversible	Reversible	
Prodrug	Yes (prodrug, CYP dependent, 2 steps)	Yes (prodrug, CYP dependent, 1 step)	No	No	No	-	
Frequency	Oral, loading dose 300/600 mg, 75 mg once daily	Oral, loading dose 60 mg, then 10 mg/5 mg daily	Oral, loading dose 180 mg, then 90 mg twice daily	IV, single dose	30 mcg/kg i.v. bolus prior to PCI followed immediately by an infusion of 4 mcg/kg/min continued for at least 2 h or for the duration of the PCI, whichever is longer	8/16 mg subcutaneous injection	
Onset of effect	2–8 h	30 min–4 h	30 min–2 h	Immediate within 2 min	Immediate: 2 min	15 min, platelet inhibition	
Interaction with CYP targeted drugs	CYP2C19	CYP3A4/CYP2B6	CYP3A4 inhibitor	-	-		
Effect lasts for	7–10 days	7–10 days	3–5 days	Completely reversed within 24 h	Till infusion	Platelet inhibition maintained for 8 h and reversible within 24 h	
Steady state IPA	40–62%	70%	80–90%	-	>90%	-	
Dose adjustment in kidney failure	No dose adjustment	No dose adjustment	No dose adjustment	No dose adjustment	No dose adjustment	No dose adjustment	
Recommended withdrawal before surgery	5 days	7 days	3–5 days	Normalization of platelet function with in 24 h	Normalization of platelet function within 60 min after discontinuation	Reversible platelet function within 24 h	

## 4. Individualization of DAPT Agent: Prasugrel vs. Ticagrelor vs. Cangrelor

After establishment of the superiority of prasugrel and ticagrelor over clopidogrel, there has been an ongoing debate regarding which one of these new agents is superior over the other. Ticagrelor has shown superiority over prasugrel in patients of older age (>75 years), lower weight (<60 kg), and history of prior stroke/TIA along with showing benefit in CV death and lesser bleeding. Ticagrelor also has shown benefit in medically managed ACS patients and ACS patients undergoing CABG. Although, ticagrelor has the additional adverse effect of dyspnea and bradycardia apart from bleeding as compared to prasugrel. Very few studies have attempted a head-to-head comparison between prasugrel and ticagrelor.

In the PRAGUE-18 study, prasugrel and ticagrelor were compared in STEMI patients undergoing primary PCI for the primary end point of death, reinfarction, urgent TVR, stroke, bleeding, or prolonged hospitalization at 7 days. The study showed similar efficacy and bleeding for either prasugrel or ticagrelor. This trial was stopped early due to futility [37].

A meta-analysis undertaken by Bundhun et al. performed a head-to-head comparison of prasugrel versus ticagrelor in patients with acute coronary syndrome. Though this meta-analysis included only 4 studies and 563 patients, it concluded that in patients with ACS, both prasugrel and ticagrelor showed similar adverse cardiovascular outcomes and bleeding events and no significant difference was observed between these two newer antiplatelet agents during the head-to-head comparison [38].

## 5. ISAR REACT 5—Game Changer or Watershed 

In the landmark ISAR-REACT study, investigators compared prasugrel and ticagrelor in STEMI, NSTEMI, and unstable angina patients undergoing PCI. The primary end point of the study was a composite of death, MI, or stroke at 12 months. The safety end point evaluated in the study was BARC type 3–5 bleeding. The study concluded that in ACS patients with or without ST-segment elevation, treatment with prasugrel as compared with ticagrelor significantly reduced the composite rate of death, myocardial infarction, or stroke (hazard ratio (HR) 1.36; 95% confidence interval (CI) 1.09–1.70; *p* = 0.0006) [5]. Definite or probable stent thrombosis was higher with ticagrelor although the value did not attain statistical significance (1.3% vs. 1.0; 95% CI 0.72–2.33). Bleeding events were higher with ticagrelor also, though not statistically significant (HR 1.12; 95% CI 0.83–1.51; *p* = 0.46)

Despite the clear verdict in favor of ticagrelor in the ISAR REACT 5 study, there were a few noteworthy limitations of the trial highlighted below. 

First, the trial data were surprising as benefits obtained with prasugrel versus ticagrelor were better than benefits obtained with prasugrel versus clopidogrel. Second, the trial had an open-label design and there was no oversight on drug adherence. Third, ticagrelor was prescribed for a median duration of 84 days vs. 120 days for prasugrel which could be reason for favorable prasugrel results. Fourth, in the ticagrelor arm the drug was given before the coronary angiogram in all while in the prasugrel arm the drug was prescribed after the angiogram in patients diagnosed with NSTE-ACS introducing heterogeneity in treatment strategies (pre-treatment vs. no pre-treatment). Other pitfalls of the study included 50% screen failure, 33% drop out rate, and lower rates of CABG (2%) vs. 10% in the PLATO trial. 

In a pre-specified analysis of the diabetic subset of the ISAR REACT 5 trial, efficacy of ticagrelor was comparable with that of prasugrel. In the trial, 22.2% had DM with similar baseline characteristics with the exception of a higher proportion of prasugrel-assigned patients that had previous CABG. The efficacy advantage of prasugrel over ticagrelor observed in the entire ISAR-REACT 5 population was confined to patients without DM, though both groups appear to be associated with a similar risk for bleeding irrespective of diabetic status [6,39]. Similarly, in the pre-specified analysis of an STEMI subset of the ISAR REACT 5 trial, there was no significant difference in the primary endpoints between prasugrel and ticagrelor [7,40]. However, ticagrelor was associated with a significant increase in the risk for recurrent myocardial infarction. However, in post hoc analysis of the NSTEMI subgroup of ISAR REACT 5, prasugrel was found to be superior to ticagrelor in reducing the combined 1-year risk of death, MI, and stroke without increasing the risk of bleeding [41].

To settle the debate over superiority, Navaresse et al. performed a network met-analysis of 52,816 patients across 12 RCTs including ISAR REACT-5 [42]. They simultaneously performed direct pairwise evaluation of safety and efficacy. Ticagrelor significantly reduced all-cause mortality and CV mortality over clopidogrel while with prasugrel the values were not significant. For overall myocardial infarction, prasugrel achieved significant reduction over clopidogrel while ticagrelor failed to do so. The authors noted a differential effect of ticagrelor on reduction in spontaneous MI rather than periprocedural MI. Stent thrombosis was significantly reduced by both drugs to the tune of 28–50%. As expected, major bleeding was increased by both drugs similarly (HR-1.26–1.27) vis-à-vis clopidogrel. For all parameters evaluated by the authors, the difference between prasugrel and ticagrelor was not significant. The authors did note the open label nature of ISAR REACT 5 compared to blinded pivotal trials PLATO and TRITON TIMI 38. They also note that higher mortality reduction with ticagrelor warrants further research as it has potential to impact public health. 

**Figure 2:**
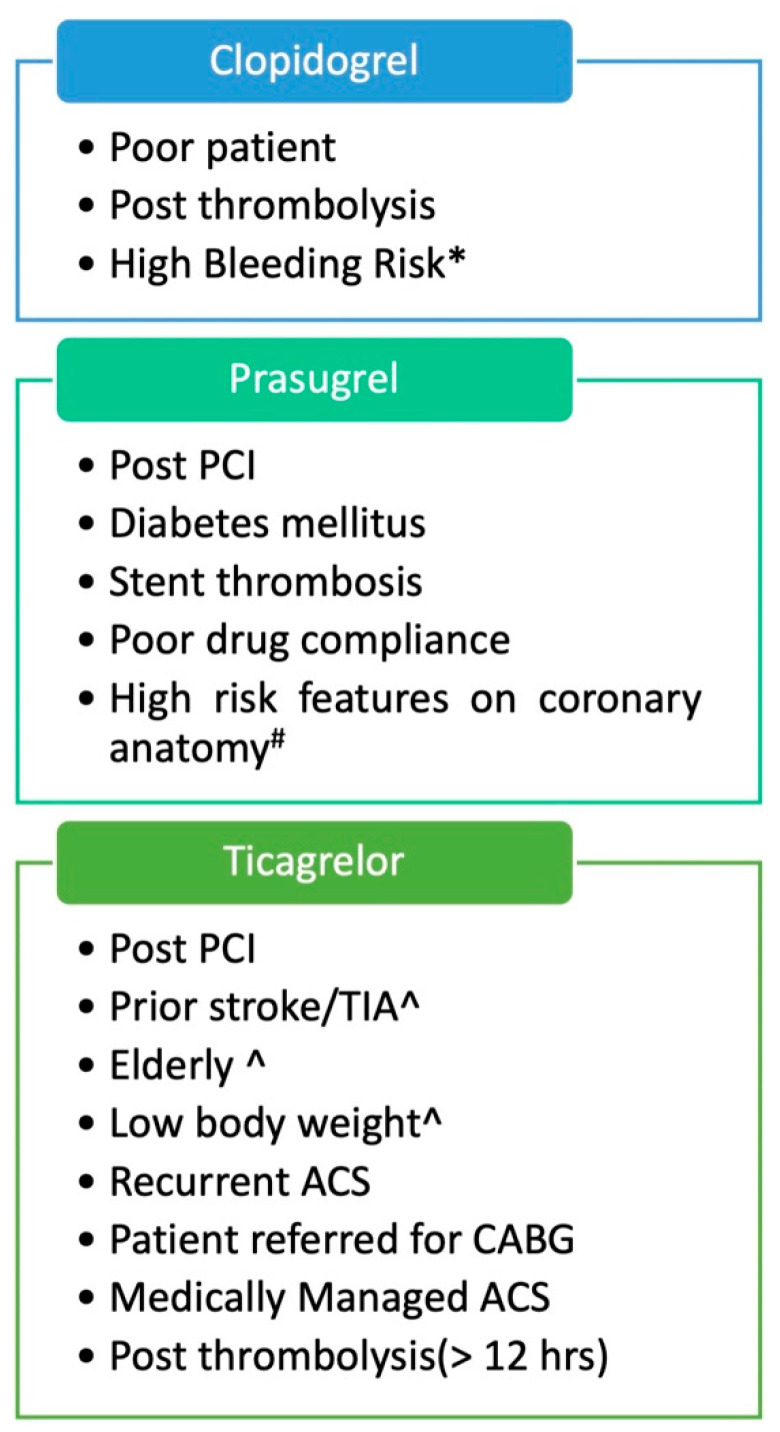
Suggested choice of P2Y12 inhibitor therapy according to clinical scenario based on the available clinical evidence. (^ A lower loading dose of 30 mg and maintenance dose of 5 mg can be utilized for prasugrel as also done in ISAR REACT-5. ^#^ Left main coronary disease, bifurcation, chronic total occlusion, sole surviving vessel. * PRECISE-DAPT or PARIS bleeding score should be used. A “De-escalation” strategy with initial course of prasugrel/ticagrelor for the first few weeks after ACS followed by clopidogrel has now been successfully tried).

Figure 2 depicts the appropriate clinical scenarios where each of these three drugs can be preferred. 

## 6. Upstream and Downstream P2Y12i Administration

Administration of P2Y12i in addition to aspirin, before performing the coronary angiography, is called pretreatment (upstream administration) while P2Y12i after the anatomy is known as downstream administration. In STEMI management, upstream administration is an established practice and found to be beneficial. However, in patients presenting with NSTEMI the practice has been debated over the years. The benefit of upstream administration includes better antiplatelet effects, more ischemic protection while waiting to undergo coronary angiography, less periprocedural thrombotic complications, and less need for bailout administration of GpIIb/IIIa inhibitors in the case of PCI while on the downside it increases bleeding and may be harmful in patients who are referred to CABG. Previous studies have shown variable results. The PCI sub-study of the CURE trial, PLATO trial supported pretreatment while the ACCOAST trial and DUBIUS trial showed no significant benefit [43,44]. Similarly, the ATLANTIC trial also failed to find superiority of ticagrelor pretreatment in STEMI [31].

## 7. P2Y12i in Special Scenarios—CKD and DM

Chronic kidney disease (CKD) represents a state of heightened cardiovascular risk and consequently cardiovascular diseases are the predominant cause of death in CKD subjects too. The incidence of ACS in the setting of renal replacement therapy can be as high as 40% [45]. Proteinuria, once developed, aggravates inflammation and alters the coagulation process contributing to the accelerated risk of ACS. Such patients with the ominous duet of ACS and CKD have extensive, calcific, and proximal CAD on angiography indicating the need for potent antiplatelet therapy [46]. However, worsening renal function is also a risk factor for bleeding, indicating the need to balance the ischemic and bleeding risk. On the other hand, previous studies have recorded a poor response to clopidogrel in patients with worsening glomerular filtration rate (GFR) and those undergoing dialysis [47,48]. In a sub-study of CKD patients from the PLATO trial, ticagrelor was superior to clopidogrel in reducing the primary end point by 23% (higher than the primary study) [49]. Ticagrelor also reduced morality while major bleeding rates were also not different from clopidogrel. More recent data from RENAMI and BLEEMACS registries also supports the use of prasugrel and ticagrelor in CKD for reduction of mortality and reinfarction rates [50,51]. The rates of major bleeding were not increased by these potent P2Y12i therapies. A recent metanalysis explored the net clinical benefit of potent P2Y12i therapy compared to standard dose clopidogrel in CKD patients across 12 studies [51]. Compared to clopidogrel-based DAPT, ticagrelor- and prasugrel-based DAPT reduced all-cause death, myocardial infarction, and MACE without increasing the risk of major bleeding. The effect on cardiac death and stent thrombosis was neutral and minor bleeding events were increased. Although, in severe CKD (eGFR < 30 mL/min) or those on dialysis both major as minor bleeding rates were higher compared to moderate CKD underscoring the need to exercise caution in this subset. 

Diabetes mellitus (DM) also represents a major risk factor of cardiovascular disease directly and indirectly being a major cause of CKD worldwide. Patients with DM with ACS have higher platelet activity, thrombotic events, and death compared to ACS without DM [52]. Interestingly, patients with DM have also reported poor responsiveness to clopidogrel therapy [53]. The OPTIMUS trial demonstrated that despite doubling clopidogrel dose antiplatelet activity remained suboptimal in more than half of patients underscoring the need for potent P2Y12i [54]. In patients with pre-existing DM in the PLATO trial, ticagrelor reduced ischemic events including stent thrombosis and all-cause mortality without increasing bleeding events [54,55]. Prasugrel reduced ischemic events to a greater extent in DM patients in the TRITON TIMI 38 study compared to non-diabetic patients (risk reduction 30% vs. 14%, respectively) [56]. There was no increase in bleeding events with DM and hence net clinical benefit was also higher with prasugrel compared to clopidogrel. In the AdHoc PCI study (troponin negative ACS patients undergoing coronary intervention), ticagrelor achieved faster and more potent platelet inhibition in DM and non-DM subjects alike [57]. In a pre-specified analysis of DM patients with ACS undergoing coronary intervention from the ISAR-REACT 5 study, there was significant interaction of diabetic status and treatment effect [39]. In patients with DM, there was no difference in efficacy between prasugrel and ticagrelor while in patients without the disease prasugrel was superior to ticagrelor. Going by the above-mentioned clauses, patient with both diabetes and CKD comprise a very high-risk subset of ACS. In this scenario too, ticagrelor reduced ischemic events over and above clopidogrel without any increase in bleeding events [58]. It is apparent from the above-mentioned arguments that potent P2Y12i are the need of the hour in ACS patients with high-risk subsets such as DM and CKD. 

## 8. Guideline Track and Pending Issues

Current treatment guidelines (AHA and ESC) recommend the use of dual oral antiplatelet therapy consisting of aspirin and a platelet P2Y12 receptor antagonist for the management of patients with ACS and/or patients undergoing PCI in order to prevent stent thrombosis and future atherothrombotic events. Ticagrelor and prasugrel are preferred P2Y12 inhibitors for ACS patients over clopidogrel owing to their superior potency and net clinical benefits [2,3]. The 2018 CCS guidelines on antiplatelet therapy has indicated certain clinical and angiographic predictors of high ischemic risk as well as bleeding risk [59]. The use of Novel P2Y12i is indicated in patients with high ischemic risk. The 2020 Asia Pacific Society of Cardiology (APSC) similarly recommends ticagrelor and prasugrel in preference over clopidogrel in ACS patients [4]. 

The search for an ideal antiplatelet agent with effective platelet inhibition without increased risk of bleeding is still ongoing. The established practice of aspirin and P2Y12 for 1 year post has also been challenged over time and now we have reached a scenario where for SAPT/DAPT/Triple therapy any of the three can be suitable based on a patient’s profile. A discussion about DAPT vs. a dual pathway inhibitor remains an open debate with role of DAPT established as standard practice. The ALTAS ACS 2-TIMI 51 trial demonstrated that addition of low-dose rivaroxaban (2.5 mg BD) over DAPT for a mean of 13 months reduced major adverse cardiovascular events. However, at the same time, it increased the rates of major bleeding but not fatal bleeding [60]. Recently, two new trials, TWILIGHT and TICO have successfully explored the role of ticagrelor monotherapy beyond three months following ACS [61,62]. 

The duration of DAPT is another area of debate. The PEGASUS TIMI 54 and DAPT studies have suggested the role of prolonged DAPT with potent P2Y12 inhibitors after ACS/PCI [63,64]. Other data showed a similar event rate of shorter DAPT as compared with longer DAPT [65,66]. All the above issues will remain in the future domain and more data is needed before any definite conclusion is reached.

## 9. Future Directions 

**Figure 3:**
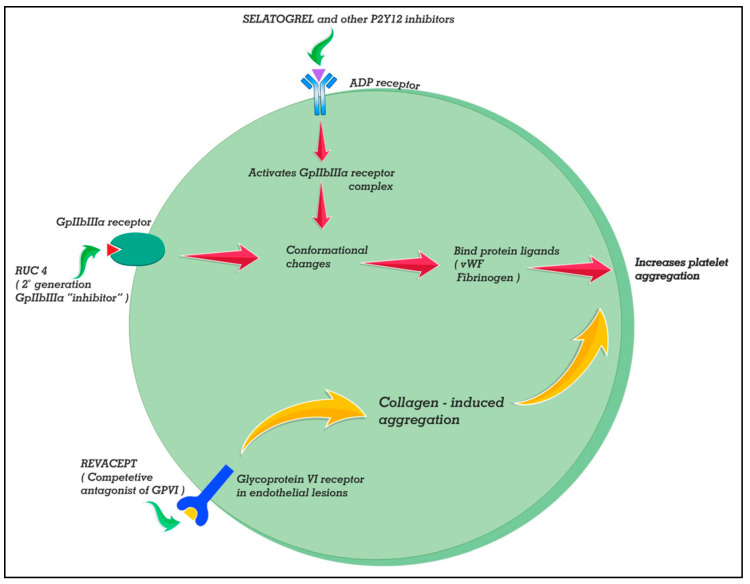
Mechanism of action of three novel antiplatelet drugs in the making. Selatogrel (ACT-246475) is a novel 2-phenylpyrimidne-4-carboxamide analogue that reversibly inhibits P2Y12 receptors. It can be administered subcutaneously for early, pre-hospital treatment of acute coronary syndrome. Revacept is a fusion molecule of extracellular domain of the GPVI receptor and human Fc-segment. It inhibits collagen-mediated platelet adhesion and aggregation selectively at the site of plaque rupture. RUC-4 is a second-generation Gp IIb/IIIa inhibitor with a subcutaneous node of administration. The drug additionally has the advantage that it does not induce antibody-induced thrombocytopenia unlike other conventional GPIIb/IIIa.

Selatogrel (ACT-246475) is novel a 2-phenylpyrimidne-4-carboxamide analogue that reversibly inhibits P2Y12 receptors (see Figure 3). The peak plasma concentrations are attained at 30 min after the 16 mg dose while with the 8 mg dose it takes 60 min. Selatogrel is being developed for subcutaneous administration for early, pre-hospital treatment of acute coronary syndrome. In stable CAD patients, a phase 2 study demonstrated prompt, potent, and consistent platelet P2Y12 inhibition sustained for >8 h and reversible within 24 h [67]. More recently, another phase 2 study in acute MI demonstrated that >90% of patients had good platelet inhibition (<100 PRU at 30 min post injection) with either 8 or 16 mg doses [68]. The subcutaneous dosing offers the prospect of easier administration in comatose patients and self dosing with a parenteral agent with remote monitoring or a resource-poor setting.

Another novel molecule, which is a competitive antagonist to collagen GPVI signaling, is entitled Revacept (AdvanceCOR GmbH, Planegg, Germany). Revacept is a dimeric, soluble fusion protein composed of the extracellular domain of the GPVI receptor and the human Fc-segment [69]. It competes with endogenous platelet GPVI for binding to exposed collagen fibers and inhibits collagen-mediated platelet adhesion and aggregation selectively at the site of plaque rupture. Because the drug is lesion directed, revacept does not interfere with the function of circulating platelets beyond the atherosclerotic lesion and hence does not even prolong bleeding time. Addition of the drug over and above aspirin, ticagrelor, and abciximab has been shown to achieve greater plaque-induced platelet inhibition both in static and flowing experimental models without increasing bleeding [70]. However, in a randomized, double-blinded placebo-controlled study (phase 2 ISAR-PLASTER trial) reduction of myocardial injury was not shown in patients with stable ischemic heart disease undergoing PCI. However, there was significant reduction (though small!) in collagen-induced platelet aggregation compared to placebo with a 160 mg dose [71]. Bleeding events defined as BARC type 2 or higher were not different between revacept and placebo. Interestingly, ADP-induced platelet aggregation was not affected by the drug.

RUC-4 is a second-generation Gp IIb/IIIa inhibitor designed for an unmet need in the acute phase of STEMI with a subcutaneous mode of administration making it ideal in prehospital settings [72]. The drug additionally has the advantage that it does not induce conformational changes in the receptor which can expose some epitopes leading to antibody-induced thrombocytopenia due to preformed antibodies in the patient unlike its previous congeners. This has been made possible because of utilization of Mg^2+^-dependent binding to the beta-3 subunit instead of the carboxyl-group-dependent binding utilized by eptifibatide or tirofiban [73]. The first-in-man phase 1 study reported that a single S/C injection provides fast, potent, and short-acting antiplatelet action [74]. The drug had excellent tolerability up to doses of 0.075 mg/kg in healthy volunteers and patients with stable CAD. A recent study has published data from an open-label, dose escalating, and phase 2 study of a weight-adjusted dose of RUC-4 in 27 patients with STEMI [75]. A single S/C dose of the drug (at different doses of 0.075, 0.090, and 0.110 mg/kg) resulted in high-grade inhibition of platelet function within 15 min. There were no episodes of thrombocytopenia at 72 h, albeit injection site reaction was not uncommon (seen in 41%).

## 10. Mitigation of Bleeding

**Figure 4:**
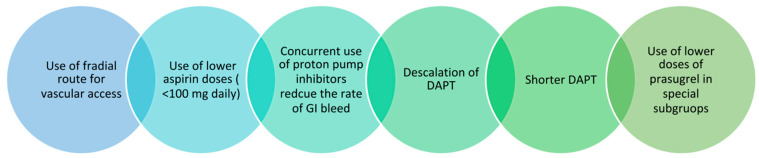
Various methods for mitigation of bleeding with the use of dual antiplatelet therapy (DAPT—dual antiplatelet therapy).

Bleeding is a concern with DAPT and more so with novel P2Y12i drugs as discussed previously. We are also now aware that bleeding following ACS/PCI is not benign and can increase mortality by 3 times. Figure 4 depicts the various approaches to limit bleeding when utilizing antiplatelet agents. Of all the points, “de-escalation” needs special mention. It refers to the short-term use of potent P2Y12i immediately after ACS for a few weeks and the switch to clopidogrel later (4 weeks or 1 month). The switch can be guided by platelet function testing (PFT) or be random. Two moderate-sized RCTs—TROPICAL-ACS and TOPICS—have demonstrated success in terms of reduction in bleeding events with both the approaches in patients with ACS [76,77]. The formed study utilized PFT at 14 days to guide de-escalation of P2Y12i therapy while the latter switched to clopidogrel at 1 month. The use of guided or unguided de-escalation will depend upon availability of PFT, ischemic risk, and clinician judgement.

Genetic testing can be utilized to identify patients with the CYP2C19 phenotype who are at risk of clopidogrel resistance and may benefit from potent P2Y12i therapy. This strategy entails reducing bleeding by selective use of prasugrel and ticagrelor in those at high risk only. The POPULAR GENETICS study tested this strategy in 2488 patients undergoing primary PCI [78]. The genotype-guided arm was non-inferior to the standard arm for thrombotic patients while significantly reducing bleeding. Similar benefits of a genotype-guided selection of prasugrel in ACS was also seen in the PHARMCLO trial [79]. Galli et al. performed a meta-analysis of 15 trials of potent P2Y12i therapy in ACS [80]. The study differs from the previous metanalysis by Navaresse et al. by additionally including RCTs utilizing a guided strategy (3 PFT based and 2 genetic based) for selecting potent P2Y12i. Compared to clopidogrel therapy, only the guided therapy arm significantly reduced MACE but not the ticagrelor arm or prasugrel arm. Bleeding was increased by both the ticagrelor arm and the prasugrel arm but not the guided strategy arm. This large meta-analysis of >61,000 patients has pitchforked guided therapy back into spotlight.

Another paradigm shift discussed previously is to switch to potent P2Y12i-based SAPT after an initial DAPT phase following ACS as explored in TWILIGT and TICO trials. A recent meta-analysis of 4 RCTs involving >20,000 patients revealed that such a short DAPT regimen was non-inferior for MACE while major bleeding was significantly reduced [81]. Caution needs to be exercised while interpreting the positive results of the study as the RCTs did not enroll patients of ACS per se but rather included patients undergoing PCI for various indications including ACS. As alluded to previously, ACS remains a subset with high ischemic risk necessitating potent and prolonged DAPT of 12 months. However, reassuring data is available from the post hoc analysis of the NSTE-ACS subset of the TWILIGT trial suggesting no heterogeneity of treatment effect [82]. The bleeding benefits were rather more pronounced.

Integration of ischemic risk and bleeding risk of the patient in decisions regarding initiation and extension of dual antiplatelet therapy is advocated by guidelines. The ESC 2017 update on antiplatelets strongly advocates for the use of DAPT and PRECISE DAPT score in decision making [83,84,85,86]. The 2018 CCS guidelines on antiplatelet therapy have indicated clinical conditions with high ischemic risk warranting novel P2Y12i like multivessel disease, multiple stents, complex bifurcation, total stent length > 60 mm, chronic total occlusion, and bioabsorbable vascular scaffold [59]

**Figure 5:**
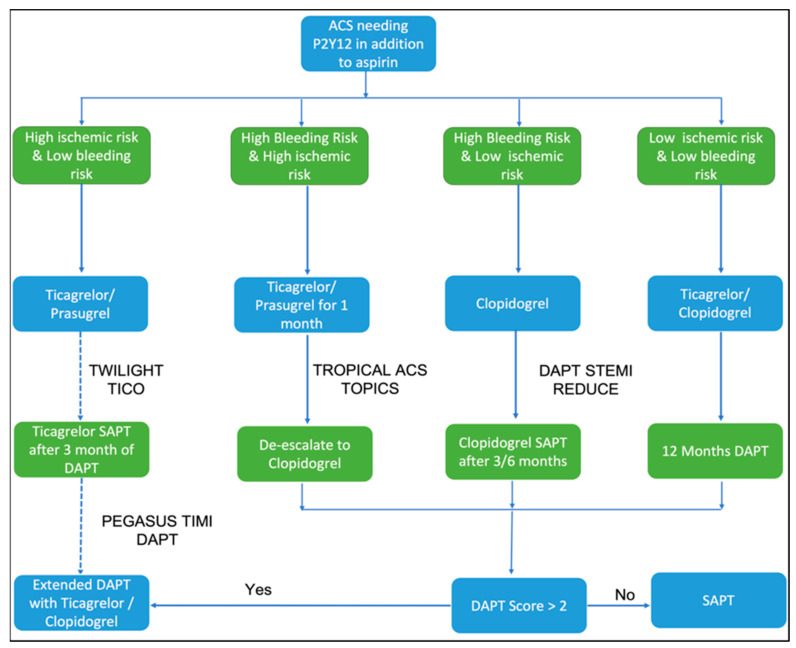
Navigating the maze of dual antiplatelet therapy after ACS based on contemporary clinical trial evidence. The DAPT score represents a combination of various demographic, clinical, and angiographic features for guiding the decision to extend DAPT beyond a year after ACS.

Figure 5 represents an algorithm for tailoring dual antiplatelet therapy incorporating the contemporary clinical evidence.

## 11. Conclusions

Management of ACS has evolved over the years but antiplatelet therapy remains at the center of management whether the patient is undergoing PCI or a conservative strategy. Clopidogrel had remained the preferred P2Y12i prior to this decade. However, over the past decade, introduction of potent P2Y12i prasugrel and ticagrelor has led to change in the P2Y12i strategy. These drugs have shown consistent reduction in ischemic endpoints over clopidogrel in large randomized trials, albeit with a marginal increase in bleeding. Current guideline recommendations prefer prasugrel and ticagrelor over clopidogrel in ACS patients. Even after a head-to-head trial, superiority of either of these drugs over another has not been demonstrated unequivocally and the ideal P2Y12i remains elusive. Bleeding does remain a concern when using newer P2Y12i and can be mitigated by taking appropriate maneuvers. The preferred P2Y12i in contemporary practice will be dependent on the patient’s clinical scenario and management strategy chosen. With three new agents in the pipeline, the unmet need in antiplatelet therapy for ACS can probably be accomplished in the near future.

## Figures and Tables

**Table 1:** The 6Cs of clopidogrel trials.

Study	Atherothrombotic Patient Type	Treatment Regimen	Primary End Points	Result/ Remark
CAPRIE (1996) [9]	Recent MI, recent ischemic stroke, or symptomatic PAD	Clopidogrel vs. aspirin	Composite of MI, ischemic stroke, or vascular death	Significant relative-risk reduction of 8.7% in the clopidogrel group (*p* = 0.043); Clopidogrel more effective than aspirin in reducing ischemic stroke, MI, or vascular death.
CURE (2001) [10]	NSTE ACS/unstable angina	Clopidogrel + aspirin vs. placebo + aspirin	Composite of CV death, MI, stroke, or refractory ischemia	Decreased death/MI/stroke by 20% in NSTE ACS/unstable angina patients; Clopidogrel in addition to aspirin has beneficial effects in patients with ACS without ST-segment elevation.
CREDO Trial (2002) [11]	Stable CAD or ACS undergoing PCI	Loading with clopidogrel 300 mg or placebo before PCI. Thereafter, all patients received clopidogrel (75 mg) through day 28. Then, day 29 through 12 months, the loading dose group received clopidogrel (75 mg daily), and the control group received aspirin throughout the study	Composite of death, myocardial infarction, or stroke at 1 year	26.9% relative risk reduction in composite endpoint in the clopidogrel group at 1 year. Clopidogrel pretreatment did not significantly reduce the combined risk of death, MI, or urgent target vessel revascularization at 28 days; Long-term (1 year) clopidogrel therapy significantly reduced the risk of adverse ischemic events.
COMMIT Trial (2005) [12]	STEMI, NSTEMI	Clopidogrel + aspirin vs. placebo + aspirin	Composite of death, reinfarction or stroke, death from any cause up to 4 week or till discharge	Significant 9% reduction in death, reinfarction, or stroke. There was also a significant 7% proportional reduction in any death; Adding clopidogrel 75 mg daily to standard treatment safely reduces major vascular events and mortality in hospital.
CLARITY-TIMI 28 (2005) [14]	STEMI	Clopidogrel + aspirin vs. placebo + aspirin in addition to standard therapy	Occluded infarct-related artery (TIMI flow grade 0 or 1) on predischarge angiogram or death or recurrent MI before angiography	Decreased death, MI, urgent revascularization by 20%. Decreased occluded artery by 36%; Addition of clopidogrel improves the patency rate of the infarct-related artery and reduces ischemic complications.
Current-OASIS 7 (2010) [17]	Acute coronary syndromes with intended early PCI	Double-dose (600 mg on day 1, 150 mg on days 2–7, then 75 mg daily) versus standard dose (300 mg on day 1 then 75 mg daily) clopidogrel, and high-dose (300–325 mg daily) versus low-dose (75–100 mg daily)	Cardiovascular death, myocardial infarction, or stroke at 30 days	Double-dose clopidogrel reduced the rate of the primary outcome (3.9% vs. 4.5%) and definite stent thrombosis (0.7% vs. 1.3%); 7 day double-dose clopidogrel regimen was associated with a reduction in cardiovascular events and stent thrombosis.

**Table 2:** Clinical evidence for tailoring DAPT based on platelet function testing.

	Study Population	Treatment Strategy	Primary End Point	Result
GRAVITAS trial (2011) [18]	Post PCI patient with drug eluting stent	After platelet function testing patients were given high-dose (150 mg daily) or standard-dose clopidogrel (75 mg daily)	Cardiovascular death, nonfatal MI or stent thrombosis at 6 months	In patients with high on-treatment reactivity after PCI with drug eluting stents, the use of high dose clopidogrel compared with standard dose clopidogrel did not reduce primary outcome
ARCTIC-GENE study (2015) [19]	Stable angina/NSTE-ACS undergoing PCI with DES implantation	Platelet function analysis in post PCI patients and clopidogrel dose adjustment	Composite of death, MI, stent thrombosis, stroke, or urgent revascularization at 12 months	No significant difference between two groups
TAILOR PCI (2020) [20]	Patients undergoing PCI for ACS or CCS	Genotype guided P2Y12 inhibitor verses conventional (no genotyping, clopidogrel in all)	Composite of cardiovascular death, myocardial infarction, stroke, stent thrombosis, and severe recurrent ischemia at 12 months	No significant difference between two groups
